# Comparison of Bispectral Index and Patient State Index values according to recovery from moderate neuromuscular block under steady-state total intravenous anesthesia

**DOI:** 10.1038/s41598-021-85419-8

**Published:** 2021-03-15

**Authors:** Doyeon Kim, Jin Hee Ahn, Gunyoung Heo, Ji Seon Jeong

**Affiliations:** 1 Department of Anesthesiology and Pain Medicine, Samsung Medical Center, Sungkyunkwan University School of Medicine, 81 Irwon-ro, Gangnam, Seoul, 06351 Korea; 2 Department of Anesthesiology and Pain Medicine, Kangbuk Samsung Hospital, Sungkyunkwan University School of Medicine, Seoul, Korea

**Keywords:** Health care, Medical research

## Abstract

There were insufficient researches of the comparison between Bispectral Index (BIS) and Patient State Index (PSI) values during the recovery of moderate NMB. We investigated the response of these indices during neuromuscular blockade (NMB) reversal by sugammadex under steady-state total intravenous anesthesia (TIVA) using propofol/remifentanil. In this prospective, observational study, patients undergoing laparoscopic cholecystectomy were enrolled. At the end of surgery, after confirming that train-of-four (TOF) count as 1 or 2, we maintained a steady state (BIS value of 40–50). After administration of 2 mg kg^−1^ sugammadex, BIS, PSI, and electromyography (EMG) signal values were recorded at one-minute intervals for 10 min. The primary outcome was the difference between the changes in BIS and PSI from baseline to a TOF ratio (TOFR) of 90 after sugammadex administration in steady-state TIVA. A total of 48 patients completed this trial. There was no significant difference between the changes in BIS and PSI values from baseline to TOFR 90 (− 0.333 ± 4.955 vs. − 0.188 ± 4.616; 95% confidence interval [CI] − 2.095 to 1.803; *p* = 0.882). Both BIS-EMG and PSI-EMG values at baseline and TOFR 90 were not statistically different (95% CI − 0.550 to 1.092; *p* = 0.510, 95% CI − 1.569 to 0.527; *p* = 0.322, respectively). No patient experienced any complications. Changes in BIS and PSI values after NMB reversal during steady-state TIVA were not significantly different. Both BIS and PSI provide trustworthy values for monitoring anesthetic depth during NMB reversal under TIVA.

**Trial Registration**: This study was registered in the Clinical Trial Registry of Korea (https://cris.nih.go.kr: KCT 0003805).

## Introduction

Electroencephalogram (EEG)-based monitoring is currently conducted to determine the depth of anesthesia. There are many EEG-based approaches such as the Bispectral Index (BIS), entropy, Patient State Index (PSI), Cerebral State Index, and Index of Consciousness^1,2^. Among these, BIS, on the basis of the frequency domain analysis, is widely used in clinical situations. In addition, PSI assists in monitoring the depth of anesthesia derived from EEG power, frequency, and phase information^3^. Both BIS and PSI values detect a burst suppression and analyze the spectrum of EEG.

Previous studies have demonstrated that electromyography (EMG) above 30 to 40 Hz together with EEG can provoke a change in BIS value due to interference^4,5^. Specifically, they reported that changes in muscle activity resulting from neuromuscular blockade (NMB) reversal affected BIS values. On the other hand, NMB abolishes patient movement and can mask inadequate anesthetic depth if a suitable monitor is not used. According to the manufacturer’s instructions, the RD SedLine EEG sensor (Masimo Corp, Irvine, CA, USA) that provides PSI values is less disturbed by EMG relative to conventional EEG monitoring equipment. This is because this system extracts EEG signals through channels separate from EMG for calculating the PSI. However, contrary to those gleaned from extensive experience with using BIS, there are relatively little data available related to PSI. In addition, to the best of our knowledge, no study examining PSI values in correlation with the recovery of NMB has been conducted to date.

We hypothesized that PSI values are less affected by NMB reversal than BIS values. Thus, the aim of the current study was to compare BIS and PSI values during recovery of moderate NMB under steady-state total intravenous anesthesia (TIVA).

## Materials and methods

### Ethics

This prospective observational trial was approved by the Samsung Medical Center Institutional Review Board (no. SMC 2018-08-021). This trial was registered in the Clinical Trial Registry of Korea (https://cris.nih.go.kr; registration no. KCT 0003805; principal investigator: Ji Seon Jeong; date of first registration: April 17, 2019) prior to recruitment of the first participant. Written informed consent was obtained from all participants before their enrollment into this study. All methods were performed in accordance with the relevant guidelines and regulations.

### Patients and anesthesia

Patients scheduled for elective laparoscopic cholecystectomy under TIVA were assessed for eligibility and included where appropriate from April 2019 to June 2019. Inclusion criteria were American Society of Anesthesiologists (ASA) physical status I or II and an age of 19 to 70 years. Exclusion criteria were body mass index of more than 30 kg m^−2^ or less than 18.5 kg m^−2^; history of allergies or hypersensitivity to propofol, remifentanil, rocuronium, or sugammadex; pregnancy; severe liver or kidney disease; neuromuscular disease; and emergency surgery.

**Figure 1:**
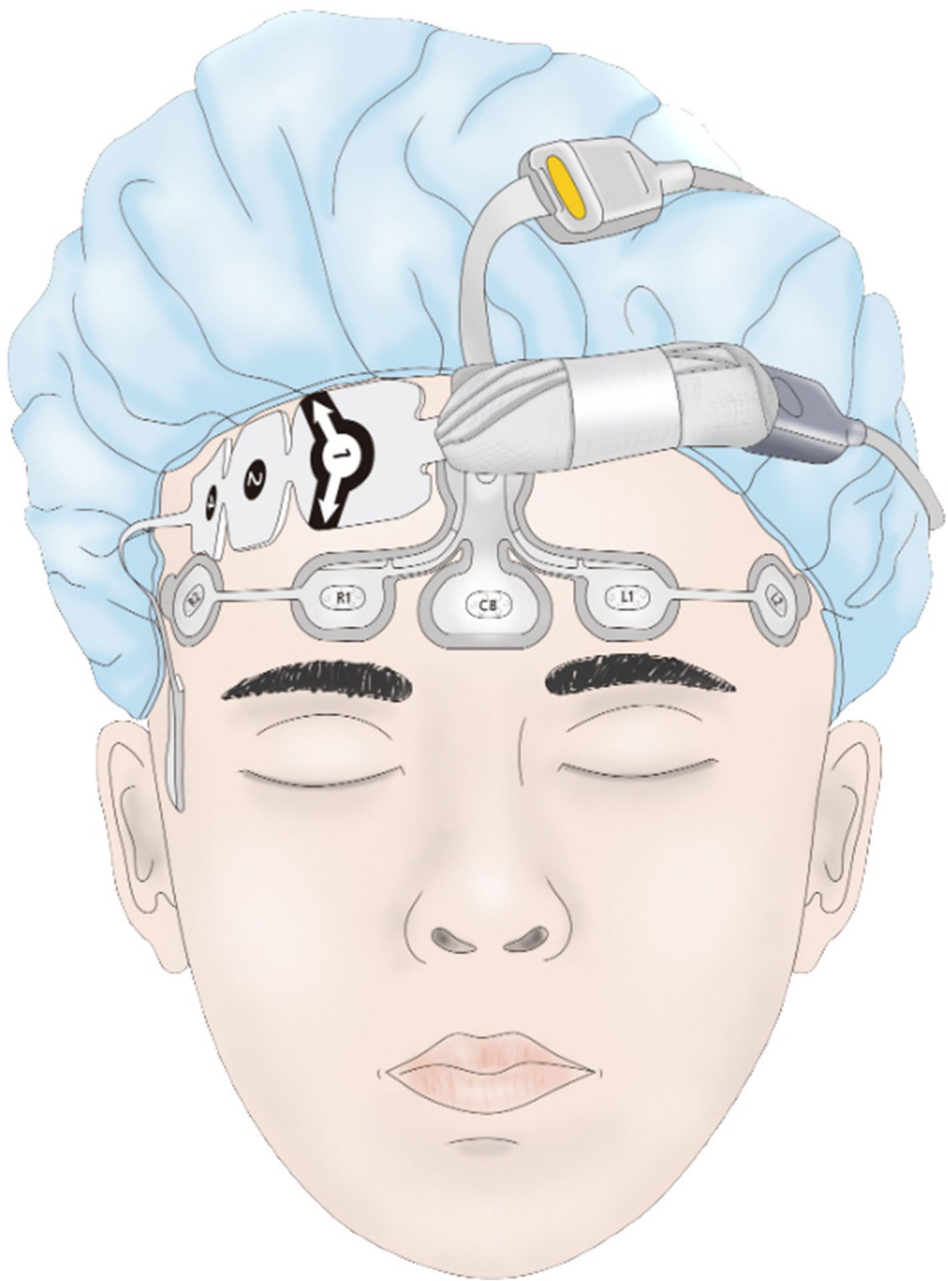
Attachment of Bispectral Index and Patient State Index sensors on a patient’s forehead.

None of the patients were given any premedication. Standard monitoring systems including electrocardiography, pulse oximetry, heart rate, and noninvasive blood pressure were applied in the operating theater. Each patient’s forehead was wiped with 70% alcohol to improve skin conductance and a BIS quarto electrode (BIS Quatro Sensors XP; Medtronic, Minneapolis, MN, USA) and PSI sensor (RD SedLine EEG Sensor; Masimo Corp., Irvine, CA, USA) were then attached to the forehead (Fig. 1). After the signal quality index was confirmed to be more than 95%, BIS and PSI values were measured from anesthesia induction to the end of anesthesia. The depth of anesthesia was adjusted based on BIS values during entire operation.

The TOF-Watch SX (Organon Ltd., Dublin, Ireland) was applied at the right adductor pollicis to monitor NMB. After five seconds of 50-Hz tetanic stimulus, the TOF-Watch SX was calibrated using the automated CAL2 mode. Patients were preoxygenated with 100% O_2_ via a facial mask and anesthesia was induced with propofol and remifentanil through target–controlled infusion (TCI). Marsh and Minto models were used to measure target effect-site concentrations (Ce) for propofol and remifentanil, respectively^6,7^. Ce was set to 5 mg mL^−1^ for propofol and 3 ng mL^−1^ for remifentanil. After the administration of 0.7 mg kg^−1^ of rocuronium and confirmation of maximum NMB with a train-of-four (TOF) count (TOFC) of 0 two times, tracheal intubation was performed. The intraoperative end-tidal carbon dioxide level was maintained between 35 and 40 mmHg. The ulnar nerve was supramaximally stimulated with the TOF mode every 15 s and maintained as TOFC 1–2. When the TOFC reached 2 or higher during surgery, 0.15 mg kg^−1^ of rocuronium was additionally injected.

### Study protocols

At the end of surgery, 0.5 mcg kg^−1^ of fentanyl was administered for postoperative pain control and the anesthetic depth was stabilized with fixed continuous infusion doses of propofol and remifentanil to a BIS value of 40 to 50 for 10 min (steady-state). After confirming a TOFC of 1 to 2, 2 mg kg^−1^ of sugammadex was administered (baseline, T0). Parameters including TOFC or TOF ratio (TOFR) and BIS and PSI values with EMG (BIS-EMG, PSI-EMG) were recorded for 10 min at one-minute intervals (T0–T10). Considering the accuracy of the values, it was used the average of 1-min interval values of BIS and PSI for analysis. Hypotension (i.e., a mean blood pressure decrease of more than 20% from the preinduction value) was treated with 5 mg of ephedrine, while bradycardia (< 50 beats/min) was treated with 0.5 mg of atropine or 0.2 mg of glycopyrrolate. If the TOFR was less than 0.9 at T10, 1 to 2 mg kg^−1^ of sugammadex was additionally given as a rescue medication. After confirming consciousness and presence of a TOFR of greater than 0.9, the patient was transferred to the postanesthesia care unit. The range suitable for general anesthesia was defined as 40–60 for BIS and 25–50 for PSI^8^. The primary outcome was the difference between BIS and PSI changes from baseline to TOFR 90 after the administration of sugammadex in steady-state TIVA. The secondary outcomes were as follows: the difference between BIS-EMG and PSI-EMG changes from baseline to TOFR 90, the relationship between BIS and PSI values, the relationship between BIS and BIS-EMG values, and the relationship between PSI and PSI-EMG values over time. In addition, we compared the frequency of BIS and PSI values outside of the range of general anesthesia during steady-state TIVA.

### Statistical analysis

In our pilot data (unpublished), the mean (standard deviation) values of changes in BIS and PSI were 5.75 (8.86) and 2.13 (1.36), respectively. To detect a statistical difference between BIS and PSI according to sugammadex administration, we used a paired t-test. With a power of 0.8 and an alpha error of 0.05, 43 patients were required to enroll. Assuming a 10% dropout rate, we planned to recruit at least 48 patients.

Continuous variables including changes in BIS and PSI were expressed as means (standard deviations) or medians (interquartile range), while normality was assessed by the Shapiro–Wilk test. Categorical variables were expressed as numbers (percentages). A paired t-test or Wilcoxon signed-rank test was used to compare BIS and PSI, BIS and BIS-EMG, and PSI and PSI-EMG at baseline and TOFR of 90 respectively, and the changes in BIS and PSI values from baseline to TOFR 90. The relationships between BIS-EMG and BIS and between PSI-EMG and PSI over time were analyzed using a generalized estimating equation. Statistical analysis was performed using SAS version 9.4 (SAS Institute, Cary, NC, USA), and *p* < 0.05 was considered to be statistically significant.

## Results

**Figure 2:**
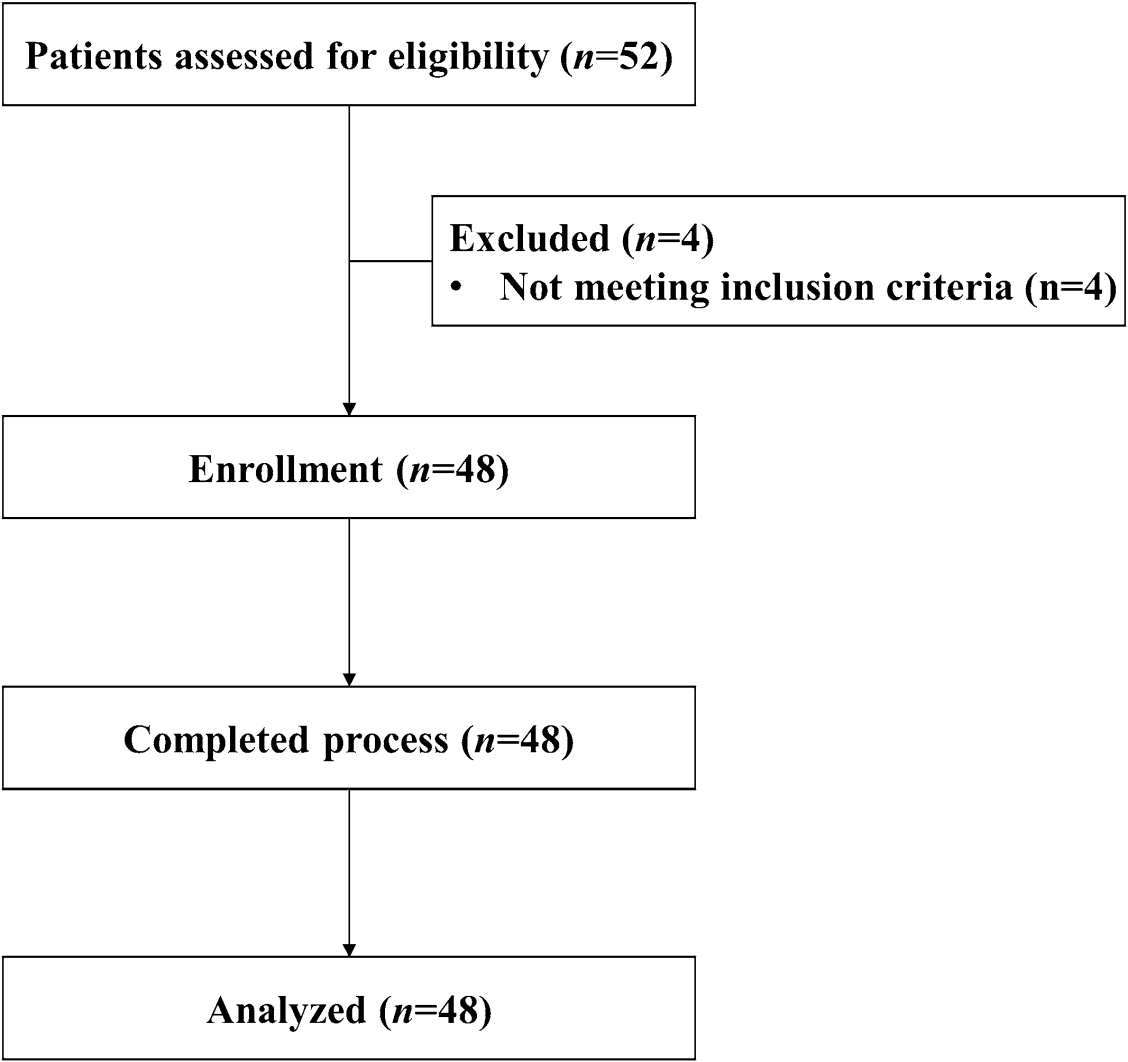
CONSORT diagram.

A total of 52 patients were screened for this trial. Among them, four cases who did not meet the inclusion criteria were excluded. Finally, 48 patients were recruited, completed the study process (Fig. 2). From T0 to T10, a total of 528 data of BIS and PSI values were acquired and were analyzed.

Patient characteristics are summarized in Table 1. At T0, 7 cases appeared TOFC 1 and 42 cases demonstrated TOFC 2. The mean (SD) of TOFC at T0 was 1.9 (0.4). BIS, PSI, BIS-EMG, and PSI-EMG values during steady-state TIVA are shown in Table 2. The mean BIS change from baseline to TOFR 90 was 0.3 (95% confidence interval [CI], − 1.1 to 1.8; *p* = 0.646). The mean PSI change from baseline to TOFR 90 was 0.2 (95% CI, − 1.1 to 1.6; *p* = 0.756). The mean difference (MD) between BIS and PSI change from baseline to TOFR 90 were not significantly different (MD, − 0.1; 95% CI, − 2.0 to 1.8; *p* = 0.896). At TOFR 90 compared to baseline, 21/48 (43.8%) patients showed increased BIS value and 22/48 (45.8%) patients showed increased PSI value. The individual BIS and PSI values by time point is shown in Supplementary Fig. S1.

**Table 1:** Patient characteristics.

Variables	Total (n = 48)
Age (yr)	53.1 ± 10.6
Gender (male)	23 (47.9%)
BMI (kg m^−2^)	24.2 ± 3.1
ASA class (I/II)	25 (52.1%)/23 (47.9%)
Anesthesia duration (min)	63.8 ± 12.3
Time to TOFR 90 (min)	1.7 ± 0.6
Effect site concentration of propofol at baseline* (μg ml^−1^)	3.0 ± 0.6
Effect site concentration of remifentanil at baseline* (ng ml^−1^)	2.0 ± 0.8
Total propofol consumption (mg)	532.1 ± 159.0
Total remifentanil consumption (mg)	0.4 ± 0.1
Total rocuronium consumption (mg)	48.1 ± 9.8

Data expressed as mean ± SD or n (%).

*BMI* body mass index, *ASA* American society of anesthesiologists, *TOFR* train of four ratio.

*Baseline was defined as the time point at which sugammadex was administered.

**Table 2:** Bispectral index (BIS) and patient state index (PSI) values during steady-state total intravenous anesthesia (TIVA).

	BIS	PSI	BIS-EMG	PSI-EMG
T0	42.1 ± 4.2	29.9 ± 5.4	32.0 ± 7.3	0.0 ± 0.0
T1	42.9 ± 5.2	30.5 ± 5.4	31.5 ± 6.8	0.0 ± 0.0
T2	42.0 ± 5.1	29.9 ± 4.5	31.8 ± 6.7	0.5 ± 3.6
T3	42.9 ± 4.9	30.8 ± 4.8	32.4 ± 6.7	0.7 ± 3.8
T4	45.1 ± 6.0	32.0 ± 6.5	32.3 ± 6.8	0.7 ± 3.9
T5	47.0 ± 7.1	32.5 ± 9.4	33.6 ± 7.6	0.7 ± 3.1
T6	47.5 ± 6.3	33.0 ± 8.4	33.1 ± 7.0	1.0 ± 3.7
T7	48.7 ± 8.8	33.5 ± 11.4	33.2 ± 6.7	1.0 ± 3.3
T8	49.3 ± 8.2	34.4 ± 9.8	33.3 ± 7.0	0.8 ± 3.3
T9	50.9 ± 9.2	34.9 ± 11.4	34.3 ± 7.2	1.2 ± 4.6
T10	51.7 ± 10.2	35.5 ± 13.9	34.1 ± 7.3	1.0 ± 4.0

Data expressed as mean ± SD.

*BIS-EMG* bispectral Index values with electromyography, *PSI-EMG* Patient State Index values with electromyography.

The mean BIS-EMG change from baseline to TOFR 90 was 0.3 (95% CI − 0.6 to 1.0; *p* = 0.510). There was no BIS-EMG effect related to BIS changes over time (95% confidence limit [CL] − 0.01 to 0.03; *p* = 0.295). The mean PSI-EMG change from baseline to TOFR 90 was 0 because the PSI-EMG signal at the baseline and TOFR 90 was not enough to have affected the processed EEG values. (95% CI − 1.6 to 0.5; *p* = 0.322). PSI-EMG did not show significant changes following PSI values over time after sugammadex injection (CL − 0.01 to 0.02; *p* = 0.67).

After sugammadex administration in steady-state TIVA, number (%) of the data out of the range for general anesthesia was 32 of 528 (6.1%) in BIS and seven of 528 (1.3%) in PSI (difference in proportion: 4.7%; 95% CI 2.5–7.2; *p* < 0.001). There were no signs of awakening and no patient showed any adverse effects during the study period.

## Discussion

Our results demonstrated that there was no difference between changes in BIS and PSI values, and the recovery of moderate NMB by sugammadex did not affect the BIS and PSI values. In addition, EMG did not have an effect on BIS or PSI over time during steady-state TIVA, and both values showed a positive correlation with each other.

Considering that BIS and PSI values, which were concurrently collected alongside EEG recordings from each device, may be affected by EMG, the administration of NMB reversal agents has the potential to cause false changes in BIS or PSI values regardless of the anesthetic depth. Previous studies have demonstrated that sugammadex and neostigmine for NMB reversal lead to an increase in both BIS and BIS-EMG^9–11^. Similarly, our previous study showed that BIS and BIS-EMG values tended to increase after NMB reversal under steady-state desflurane anesthesia^12^. On the contrary, Illman et al. reported that sugammadex does not influence the depth of anesthesia during steady-state TIVA^13^. Our results also showed that there was no change in BIS, PSI, BIS-EMG, or PSI-EMG during steady-state TIVA after sugammadex administration. Several possible theories can explain why BIS and PSI values remained unchanged in steady-state TIVA after sugammadex administration. First, as Dahaba et al. demonstrated, the degree of EMG activity after NMB reversal may differ according to whether EMG was activated before the administration of an NMB reversal agent^5^. In our study, the BIS-EMG activity prior to sugammadex administration was high in only 10 of 48 patients, and no PSI-EMG activity was expressed. Second, it may be related to the degree of NMB and the reversal of NMB that occurs after injection of the NMB reversal drugs. Le Guen et al. suggested that the activation of muscle mass was different due to the difference in sugammadex injection dose^11^. Contrary to the current study and Ilman et al.’s, they used relatively high dose of sugammadex (2 mg kg^−1^ vs 4 mg kg^−1^) and suggested that the difference of activated muscle mass caused a difference in the degree of BIS after NMB reversal. Since we induced NMB reversal in the moderate NMB of TOFC 1–2, the effect on the central nervous system was relatively small. Consequently, the changes in BIS, PSI and EMG may have been insignificant. Lastly, it is worth noting that high doses of remifentanil do not alter BIS values in the TCI mode of propofol/remifentanil anesthesia^14,15^. Compared to other intravenous and volatile anesthetics, opioids have less electrophysiological effects on the cerebral cortex. However, continuous administration of remifentanil suppressed EMG activity in steady-state anesthesia^16^ and results of the subcortical structures related to the effects of opioids are less likely to be detected in EEG^14,17^. To maintain steady-state TIVA, an average of 1.99 ng mL^−1^ of remifentanil was administered until the end of the current study. Although the amount was relatively small, the possibility of opioids affecting the EMG and EEG findings cannot be completely ruled out.

Both BIS and PSI measure the depth of anesthesia through frontal EEG analysis using numerical ranges from 0 to 100. Whereas BIS only monitors EEG in one hemisphere with a sensor attached, PSI encompasses EEG measurements in both hemispheres. Moreover, PSI has a larger number of places where sensors need to be attached to the forehead than BIS. Thus, to obtain the correct value, PSI may require more attention than BIS. According to Chen et al.^18^, BIS and PSI values showed good correlation during induction and emergence from desflurane anesthesia. Further, this was true not only in the context of desflurane anesthesia but also with other anesthesia methods (e.g., sevoflurane, sevoflurane/remifentanil, propofol, and propofol/remifentanil)^8,19,20^. However, in our study, BIS and PSI values had a weak positive correlation and the mean BIS value was about 14 higher than the mean PSI value during the study period. It is assumed that this variation relative to other studies occurred because we investigated parameters only in steady-state TIVA. Since both the BIS and PSI values we measured were within the area suitable for general anesthesia, the range of comparative values was narrower than in the aforementioned studies. In addition, there was a time lag between an EEG change and the recording of the values that reflected it. BIS and PSI devices require at least 15 and 25 s to calculate and present an average, respectively^20^.

Even if the monitor shows BIS or PSI values within a range suitable for general anesthesia, the interpretation and clinical application of those values require attention. A previous study reported that some patients experienced anesthesia awareness during propofol/remifentanil anesthesia in the range of BIS values of 50 to 60^21^. Although there was no description of the putative mechanism, the authors suggested that comparable BIS values do not assure equivalent levels of consciousness under general anesthesia. In addition, Schneider et al. demonstrated that both BIS and PSI values may be insufficient to accurately measure the level of consciousness during anesthesia^22^. At the time of anesthesia induction or surgical incision, some clinical conditions (e.g. hypoglycemia, hypovolemia, hypothermia, or hyperthermia) and the use of electric devices are known to be factors that affect BIS^23^. The impact of these factors may be common when applying instruments that measure anesthesia depth using EEG and EMG. Since the effects of various environmental factors on BIS may be different, we conducted a study in steady-state TIVA with no external stimulus. Nevertheless, the cases out of the ranges of appropriate BIS and PSI values for general anesthesia totaled 6% and 1%, respectively, although no anesthesia awareness occurred. It can be indicated that PSI reflects the depth of anesthesia relatively well in steady-state TIVA.

We acknowledged several limitations in this study. First, due to differences inherent in the algorithm to calculate each value, there was no direct comparison between BIS and PSI in the current study. To correct this difference, we compared the changes that occurred between baseline and TOFR 90. Second, since BIS only allows for EEG recording on one side of the cerebral hemisphere, comparing the PSI and the opposite-side BIS value may yield different results and even significant discrepancies, especially in patients with differences in both cerebral blood flows. However, patients with diseases such as brain injury, Alzheimer’s disease or other dementia, or cerebral palsy that can provoke a difference in cerebral blood flow did not participate in the current study. Lastly, although we consistently maintained the steady-state of TIVA during study period, it is not a common practice method in clinical anesthesia. The steady-state of TIVA was the condition that was designed for an accurate comparison of the BIS and PSI values in the current study. Thus, it needs caution to apply our results in practice.

In conclusion, changes in BIS and PSI values by the reversal of NMB during steady-state TIVA were not different nor significantly affected by EMG over time. In steady-state TIVA, the effects of EMG artifacts remain almost unaffected by an NMB reversal after sugammadex injection, while both BIS and PSI values representing the depth of anesthesia are relatively reliable.

## Supplementary Information

Supplementary Figure.

## Data Availability

The datasets generated during and/or analysed during the current study are available from the corresponding author on reasonable request.

## Abbreviations

BIS: Bispectral Index
PSI: Patient State Index
NMB: Neuromuscular blockade
TIVA: Total intravenous anesthesia
TOF: Train-of-four
EMG: Electromyography

## References

[1] Bruhn J, Myles PS, Sneyd R, Struys MM (2006). Depth of anaesthesia monitoring: What's available, what's validated and what's next?. Br. J. Anaesth..

[2] Revuelta M (2008). Validation of the index of consciousness during sevoflurane and remifentanil anaesthesia: A comparison with the bispectral index and the cerebral state index. Br. J. Anaesth..

[3] Rampil IJ (1998). A primer for EEG signal processing in anesthesia. Anesthesiology.

[4] Bruhn J, Bouillon TW, Shafer SL (2000). Electromyographic activity falsely elevates the bispectral index. Anesthesiology.

[5] Dahaba AA (2012). Effect of sugammadex or neostigmine neuromuscular block reversal on bispectral index monitoring of propofol/remifentanil anaesthesia. Br. J. Anaesth..

[6] Minto CF (1997). Influence of age and gender on the pharmacokinetics and pharmacodynamics of remifentanil. I. Model development. Anesthesiology.

[7] Marsh B, White M, Morton N, Kenny GN (1991). Pharmacokinetic model driven infusion of propofol in children. Br. J. Anaesth..

[8] Soehle M (2010). Patient state index vs bispectral index as measures of the electroencephalographic effects of propofol. Br. J. Anaesth..

[9] Vasella FC, Frascarolo P, Spahn DR, Magnusson L (2005). Antagonism of neuromuscular blockade but not muscle relaxation affects depth of anaesthesia. Br. J. Anaesth..

[10] Aho AJ (2012). Elevated BIS and Entropy values after sugammadex or neostigmine: An electroencephalographic or electromyographic phenomenon?. Acta Anaesthesiol. Scand..

[11] Le Guen M (2020). Reversal of neuromuscular blockade with sugammadex during continuous administration of anaesthetic agents: A double-blind randomised crossover study using the bispectral index. Anaesthesia.

[12] Kim D, Ahn JH, Jung H, Choi KY, Jeong JS (2019). Effects of neuromuscular blockade reversal on bispectral index and frontal electromyogram during steady-state desflurane anesthesia: A randomized trial. Sci. Rep..

[13] Illman H, Antila H, Olkkola KT (2010). Reversal of neuromuscular blockade by sugammadex does not affect EEG derived indices of depth of anesthesia. J. Clin. Monit. Comput..

[14] Koitabashi T, Johansen JW, Sebel PS (2002). Remifentanil dose/electroencephalogram bispectral response during combined propofol/regional anesthesia. Anesth. Analg..

[15] Yufune S, Takamatsu I, Masui K, Kazama T (2011). Effect of remifentanil on plasma propofol concentration and bispectral index during propofol anaesthesia. Br. J. Anaesth..

[16] Aho AJ, Yli-Hankala A, Lyytikainen LP, Jantti V (2009). Facial muscle activity, response entropy, and state entropy indices during noxious stimuli in propofol-nitrous oxide or propofol-nitrous oxide-remifentanil anaesthesia without neuromuscular block. Br. J. Anaesth..

[17] Eaan TD, Muir KT, Stanski DR, Shafer SL (1996). Using the electroencephalographic (EEG) fingerprint to define the clinical pharmacodynamics (PD) of a novel opioid: Application to remifentanil (REMI). Clin. Pharmacol. Ther..

[18] Chen X (2002). A comparison of patient state index and bispectral index values during the perioperative period. Anesth. Analg..

[19] Schneider G (2004). EEG-based indices of anaesthesia: Correlation between bispectral index and patient state index?. Eur. J. Anaesthesiol..

[20] Soehle M (2008). Comparison between bispectral index and patient state index as measures of the electroencephalographic effects of sevoflurane. Anesthesiology.

[21] Schneider G (2002). Bispectral Index (BIS) may not predict awareness reaction to intubation in surgical patients. J. Neurosurg. Anesthesiol..

[22] Schneider G, Gelb AW, Schmeller B, Tschakert R, Kochs E (2003). Detection of awareness in surgical patients with EEG-based indices-bispectral index and patient state index. Br. J. Anaesth..

[23] Dahaba AA (2005). Different conditions that could result in the bispectral index indicating an incorrect hypnotic state. Anesth. Analg..

